# A novel off-the-shelf single-fenestrated stent graft for emergent complex aortic aneurysm repair

**DOI:** 10.1016/j.jvscit.2023.101362

**Published:** 2023-10-30

**Authors:** Maysam Shehab, Gisli Gunnar Jónsson, Marek Kuzniar, Kevin Mani, David Lindström, Anders Wanhainen

**Affiliations:** a Division of Vascular Surgery, Department of Surgical Sciences, Uppsala University, Uppsala, Sweden; b Division of Surgery, Department of Surgical and Perioperative Sciences, Umeå University, Umeå, Sweden

**Keywords:** Juxtarenal AAA, Off-the-shelf stent graft, Single fenestration, Superior mesenteric artery, Thoracoabdominal aortic aneurysm

## Abstract

The off-the-shelf single fenestrated stent graft is based on the Cook Zenith fenestrated platform (Cook Medical Europe) with a premade 8-mm fenestration for the superior mesenteric artery (SMA). The device is suitable for emergency treatment of paravisceral aneurysms when combined with in situ laser fenestration for the renal arteries (and, if required, the celiac trunk). The presence of a premade SMA fenestration results in minimal visceral ischemia time. We present the case of a 69-year-old woman with a ruptured Crawford type I thoracoabdominal aortic aneurysm and a tandem abdominal aortic aneurysm that was treated successfully using the single fenestrated device with in situ laser fenestration for the renal arteries, with no SMA ischemia time. A 6-month computed tomography angiogram showed patent renovisceral stents without an endoleak.

For ruptured abdominal aortic aneurysms (AAAs) with suitable anatomy, endovascular aneurysm repair (EVAR) is the recommended standard of care.^1^ However, for emergent paravisceral aneurysms for which standard EVAR is not applicable, more complex endovascular solutions are necessary. Yet, anatomic constraints can often exclude the use of readily available off-the-shelf devices.^2^ Alternative techniques, such as physician-modified endografts (PMEGs) and chimney EVAR, have their own set of challenges and limitations.

In situ laser fenestration (ISLF) has demonstrated promising short- and mid-term results in these difficult cases.^3^, ^4^, ^5^, ^6^, ^7^ However, reducing the revascularization time of the visceral arteries is a paramount challenge to reduce the risk of ischemia-related complications.

To overcome this problem, we have customized a single fenestrated device, based on the Cook Zenith fenestration platform (Cook Medical Europe), for urgent cases to provide continual perfusion to the superior mesenteric artery (SMA) throughout the procedure during ISLF for the other renovisceral arteries.

We present a case using this novel technique to treat a patient with a ruptured type I thoracoabdominal aortic aneurysm (TAAA).

## Case report

**Fig 1:**
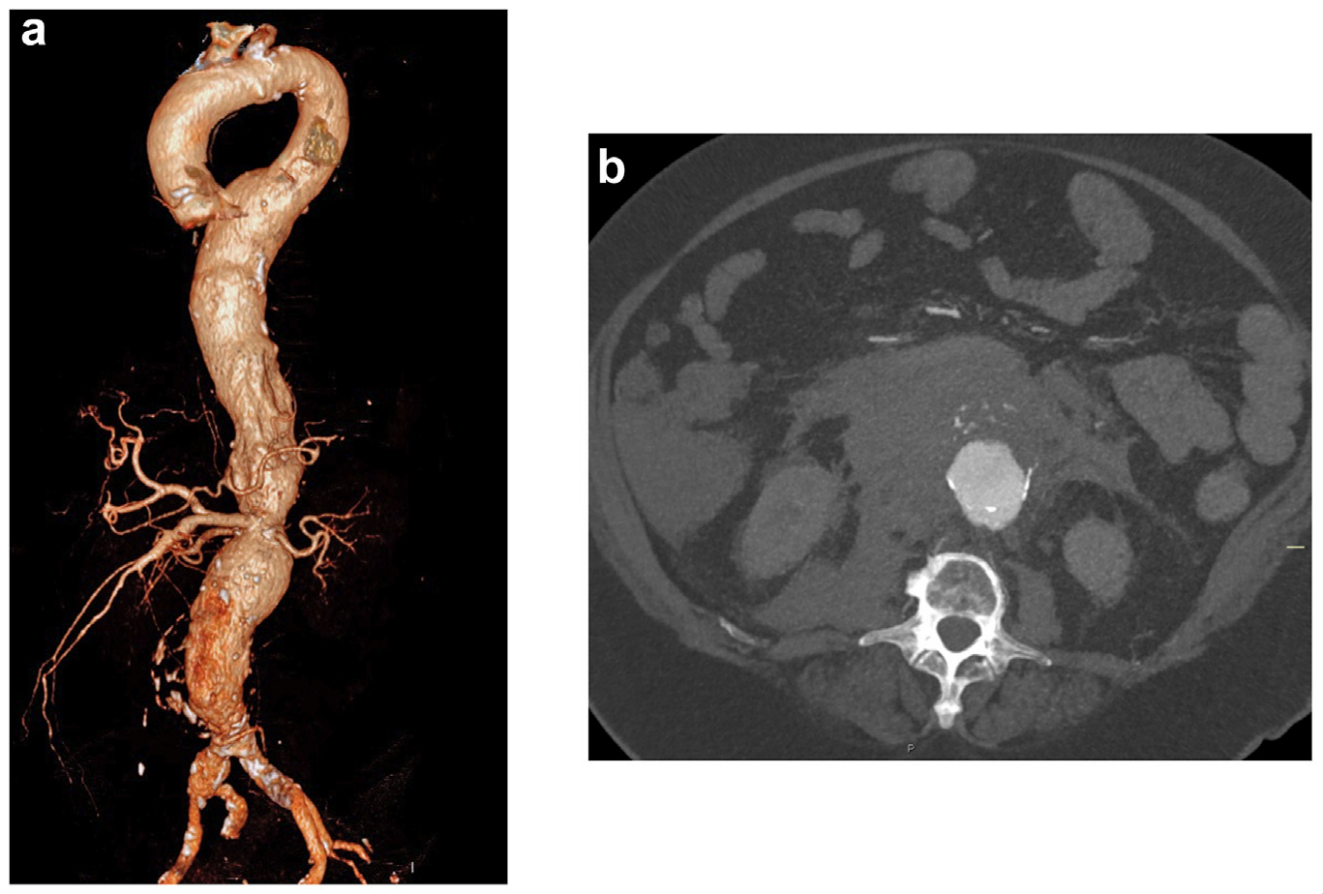
**a,** Three-dimensional reconstruction of computed tomography scan demonstrating a Crawford type I thoracoabdominal aortic aneurysm (TAAA) and a concomitant tandem abdominal aortic aneurysm (AAA). **b,** Axial view of computed tomography angiogram demonstrating the ruptured aortic aneurysm.

**Fig 2:**
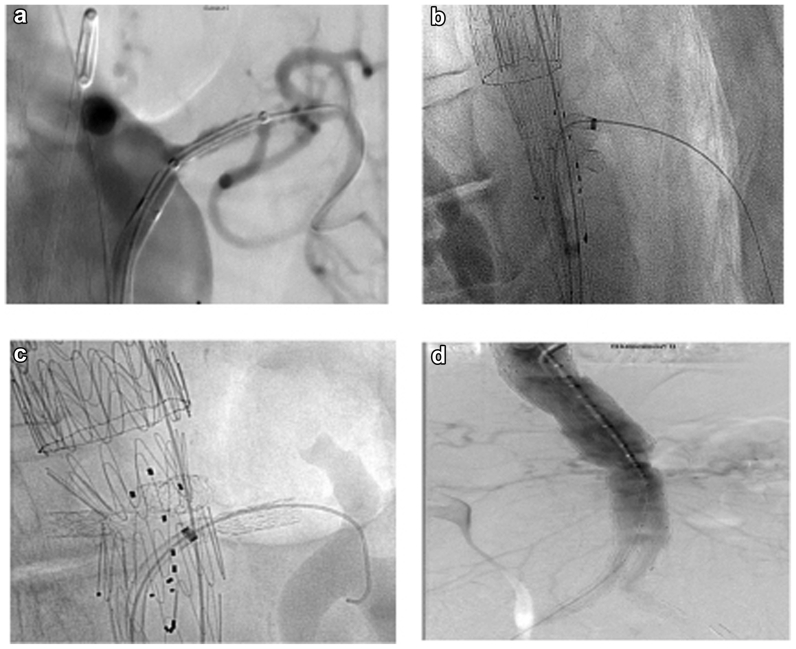
**a,** Cannulation and prestenting of the left renal artery. **b,** Cannulation of the superior mesenteric artery (SMA) fenestration and advanced steerable sheath. **c,** The tip of the steerable sheath was aligned with the ostium of the left renal artery. Next, an antegrade fenestration was performed by activating the laser probe for a few seconds, followed by advancement of a wire into the artery. **d,** Completion angiography, no early leakage nor flow compromise into the renal arteries.

A 69-year-old women with a history of type 2 diabetes mellitus and pulmonary fibrosis presented with acute abdominal pain and an episode of syncope. Her initial vital signs showed a systolic blood pressure of 105 mm Hg. An electrocardiogram exhibited a normal sinus rhythm without ischemic changes. Physical examination revealed a pulsatile tender abdominal mass. Laboratory results showed a hemoglobin level of 8.9 mg/L and creatinine of 119 μmol/L. Computed tomography angiography confirmed the presence of a ruptured Crawford type I aneurysm and a tandem large AAA with the rupture located in the pararenal segment (Fig 1). Although the SMA and renal arteries were patent, the celiac trunk was occluded. Due to a narrow pararenal aortic segment, which made standard off-the-shelf branched devices unsuitable, we opted for a single fenestrated device. The single fenestrated device is available as a premade off-the-shelf device in our institution. She was immediately transferred to the hybrid operating theater for expedited intervention. Under general anesthesia, bilateral ultrasound-guided percutaneous common femoral artery cannulation was performed, and a five-step procedure was initiated. Aortic balloon occlusion was not required due to her hemodynamic stability, and we maintained permissive hypotension until the aneurysm was excluded (Fig 2). The patient provided written informed consent for the report of her case details and imaging studies.

### First step—thoracic EVAR

A 32-200 mm conformable TAG thoracic endoprosthesis device (W.L. Gore & Associates) was implanted, with a proximal landing zone just distal to the left subclavian artery origin and extended distally with another 34-200 mm conformable TAG thoracic endoprosthesis, which landed distally just above the SMA.

### Second step—prestenting the renal arteries

Both renal arteries were cannulated and prestented with 6 × 16-mm covered balloon expandable stents (Advanta V12; Atrium Europe BV) to serve as a guide during the in situ fenestration.

### Third step—fenestrated EVAR

A single fenestration stent graft with a premade 8-mm fenestration for the SMA was implanted. An 8 × 32-mm bridging stent graft (Advanta Atrium V12) was implanted, and the fenestration was flared with a 12 × 20-mm balloon at low pressure.

### Fourth step—ISLF to the renal arteries

A 2.3-mm laser catheter (Turbo Elite; Philips Healthcare) was inserted into a steerable sheath (7.5F, 55-mm Aptus; Medtronic). The tip of the steerable sheath was adjusted to align with the ostium of the right renal artery, using the preimplanted stent as a guide. Subsequently, antegrade ISLF was performed by activating the laser probe for 1 second. After creating the laser perforation, a 0.018-in. wire was advanced into the renal artery, and a 4 × 40-mm balloon was passed along the wire to enlarge the fenestrated orifice. The 0.018-in. wire was exchanged for a 0.035-in. Rosen wire guide (Cook Medical), and the fenestration was bridged with a 6 × 22-mm Advanta Atrium V12 stent graft. The same procedure was performed to the left renal artery.

### Fifth step—EVAR and completion control

A bifurcated Zenith universal distal body endovascular graft (Cook Medical Europe) was deployed. Completion angiography showed patent visceral bridging stents and no type I or III endoleak. The total renal ischemia time was ∼25 minutes for the right kidney and ∼45 to 50 minutes for the left kidney. The patient remained hemodynamically stable with sufficient urine output during the entire procedure. Heparin was administered initially at a dose of 5000 U, with additional doses during the procedure determined by the activated clotting time. The heparin effect was reversed with protamine at the end of the surgery. The access sites were closed with fascial sutures. The total operating time was 180 minutes.

### Outcome

Postoperatively, the patient had loss of motor function in her legs, with well-perceived pulses. Despite intensive treatment with cerebrospinal fluid drainage, blood pressure elevation, and ensuring oxygenation, no improvement in leg motor function was observed. A computed tomography scan of the brain did not reveal any signs of subarachnoid hemorrhage. Subsequently, the patient was transferred from the intensive care unit to the neurorehabilitation department for continued rehabilitation.

**Fig 3:**
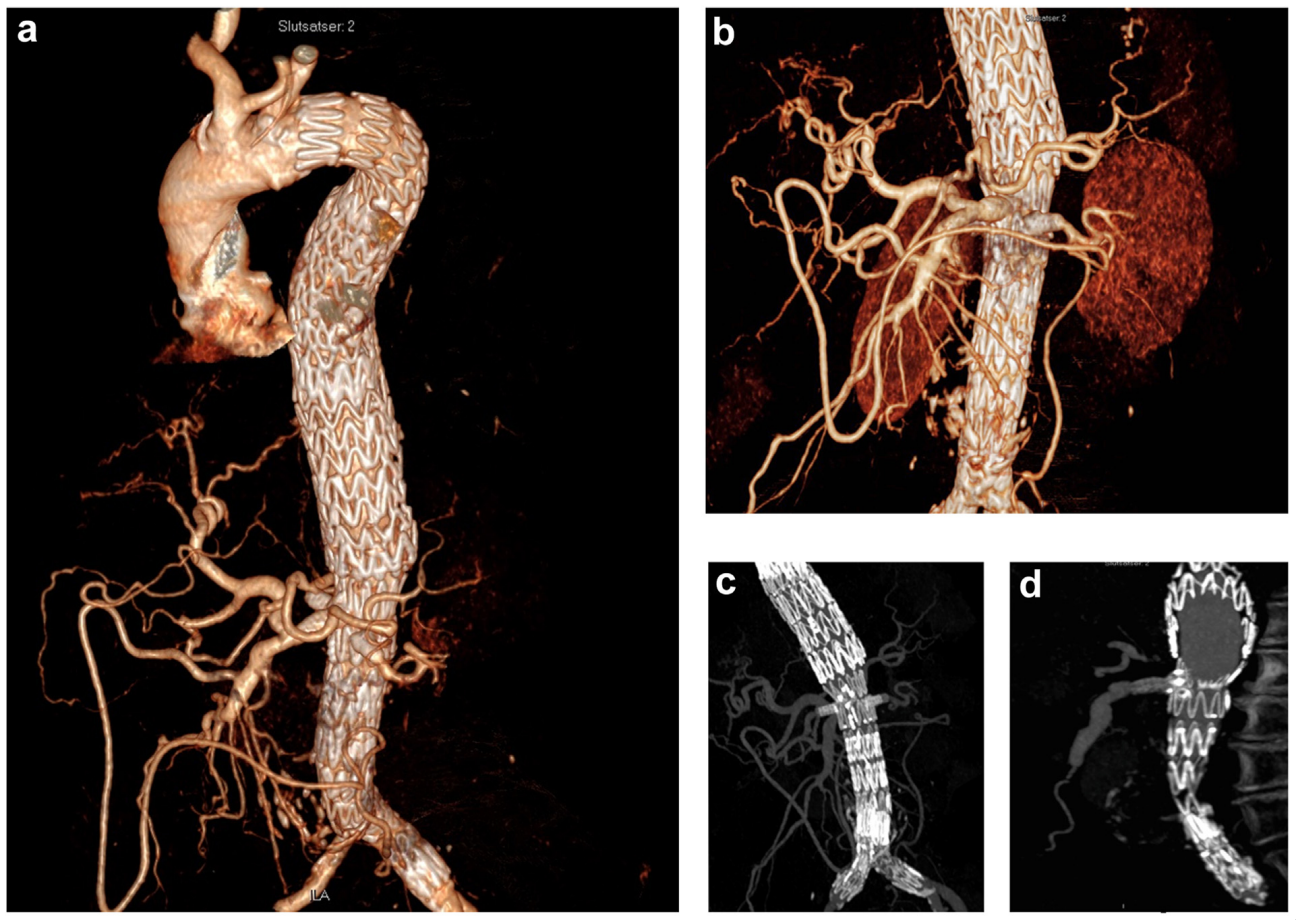
Six-month follow-up computed tomography scans. **a,** Three-dimensional reconstruction of computed tomography scan demonstrating the aortic metal jacket and stents to the superior mesenteric artery (SMA) and renal arteries. **b,** Three-dimensional reconstruction of computed tomography scan demonstrating the visceral segment. **c,** Coronal view of computed tomography angiogram showing patent renal artery stents. **d,** Sagittal view of computed tomography angiogram showing a patent SMA stent.

Six months later, the patient returned to the outpatient clinic for a routine examination. Cognitively, she was doing well, maintaining good mental acuity; however, her paraplegia remained established. A 6-month follow-up computed tomography scan depicted patent visceral stents, a thrombosed aneurysm sac without an endoleak, and a stable aneurysm diameter (Fig 3).

## Discussion

Ruptured TAAAs account for 5% to 10% of TAAAs,^8^, ^9^, ^10^ with a 30-day mortality rate of 17% to 27% after endovascular treatment.^11^, ^12^, ^13^ Etz et al^14^ reported a 19% rate of paralysis in thoracic EVAR procedures. The risk significantly increases with Crawford type II aneurysms and extensive coverage.^14^, ^15^, ^16^ Le Houérou et al^7^ shared satisfactory outcomes for nondeferrable treatment of aortic pathologies involving the visceral segment using ISLF. The technical success rate was 97%, the median SMA and renal artery ischemic time was 7 and 48 minutes, respectively, and stent-related reintervention-free survival at 1, 2, and 3 years was 94.4%, 90.6%, and 74.8%, respectively.^7^

By using an off-the-shelf, single fenestrated custom device, we mitigated the risk of mesenteric ischemia arising from SMA coverage and reduced the potential for renovisceral ischemia. This stent graft's anatomic constraints differ partially from standard designs. Although it manages severe angulation, care is essential for small or proximate target vessels. Optimal results require fenestration wall contact.

**Fig 4:**
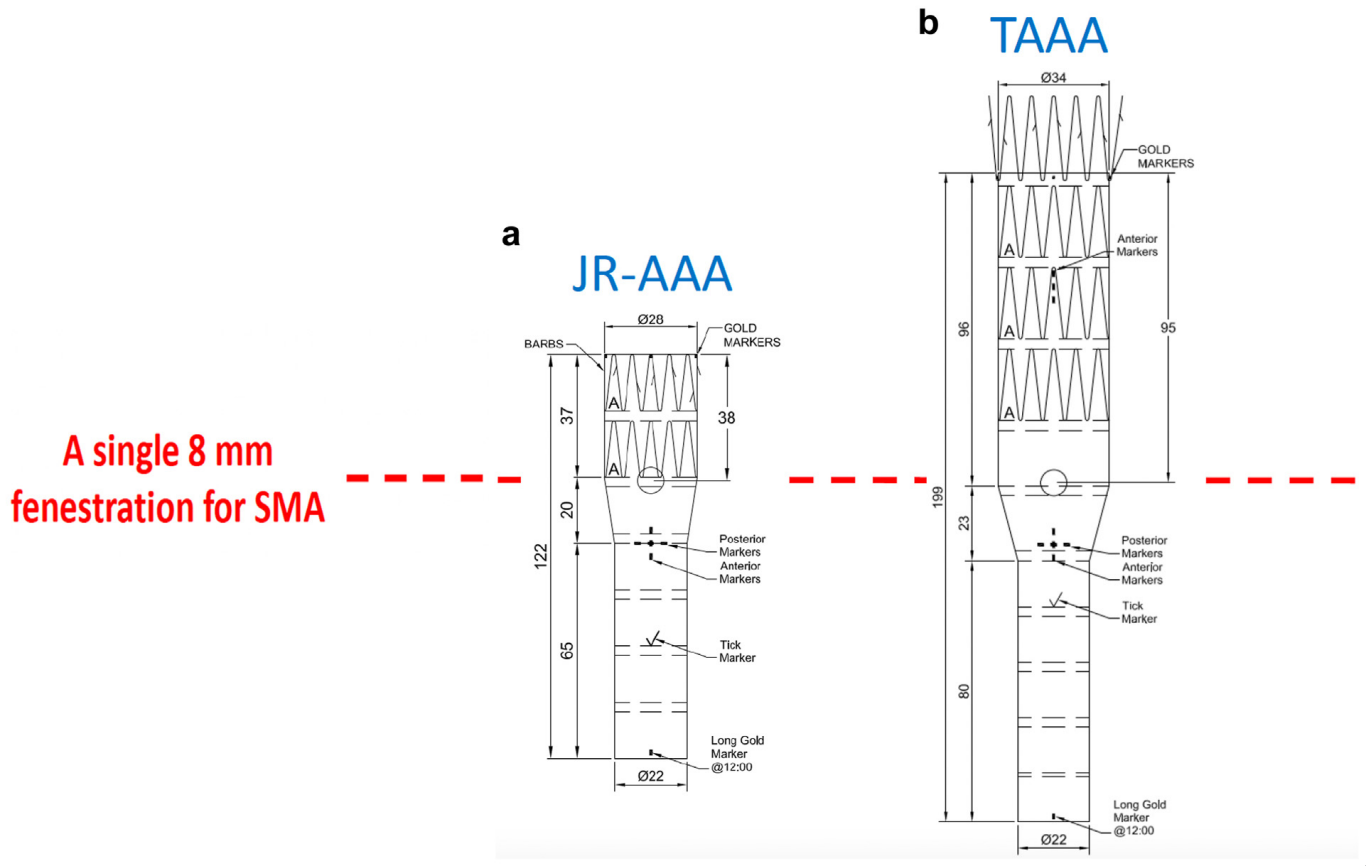
A single fenestration company-manufactured device stent graft with a premade 8-mm fenestration for the superior mesenteric artery (SMA) designed for thoracoabdominal aortic aneurysms (TAAAs). **a,** Design intended for juxtarenal abdominal aortic aneurysms (*JR-AAAs*). **b,** Design intended for TAAAs. This stent graft was used for our patient.

We currently have two different sizes of a prototype single fenestrated device with a proximal outer diameter of 28 and 34 mm. One is intended for juxtarenal aortic aneurysms (Fig 4, *a*), and the other for TAAAs (Fig 4, *b*), with the aim of avoiding unnecessary aortic coverage and reducing the risk of potential spinal ischemia. In the present case, the stent graft had a 7.7-mm outer diameter, a lower profile than that of other off-the-shelf devices. An ongoing work aims to determine the ideal specifications for a single fenestration off-the-shelf solution for most complex AAAs. Furthermore, the SMA fenestration should probably be positioned higher to avoid ISLF of the renal vessels in the tapering segment of the stent graft.

Combining this design with PMEG is possible; however, the advantage of flexible device placement would be lost. Currently, no evidence is available suggesting that PMEG fenestration outperforms ISLF in strength, durability, or patency.^7^^,^^17^

We recommend revascularizing the celiac trunk, especially with unclear SMA collateral vessels. However, in cases of circulatory instability or significant stenosis, the celiac trunk can be intentionally sacrificed.

The devices are compatible with standard devices for proximal and distal extension according to patent-specific anatomy. A study aiming to optimize the design to fit >90% of juxtarenal and pararenal aneurysms is currently underway at our center.

## Conclusions

The off-the-shelf single fenestrated stent graft is an innovatively designed stent graft with a premade fenestration for the SMA with intended ISLF for the renal arteries and, if required, for the celiac trunk. It allows for emergency endovascular treatment of complex aortic aneurysms involving the renovisceral segment. This distinctive design completely avoids ischemia for the SMA and shortens the ischemic time for the kidneys.

## Disclosures

None.
